# Efficient macrophage infection by phagocytosis of dying HIV-1 -infected CD4+T cells

**DOI:** 10.1186/1742-4690-8-S2-O31

**Published:** 2011-10-03

**Authors:** Fedde Groot, Rebecca A Russell, Amy E Baxter, Sonja Welsch, Christopher JA Duncan, Christopher Willberg, Christina Ochsenbauer, John C Kappes, Michael Shaw, Quentin J Sattentau

**Affiliations:** 1 The Sir William Dunn School of Pathology, University of Oxford, OX1 3RE, UK; 2 Structural and Computational Biology, EMBL, D-69117 Heidelberg, Germany; 3 The Weatherall Institute of Molecular Medicine, Nuffield Department of Medicine, University of Oxford, JR Hospital, OX3 9DU, UK; 4 Department of Medicine, University of Alabama at Birmingham, Alabama, 35294, USA

## Background

Macrophages are scavengers of the innate immune system that eliminate dead and dying cells, and pathogens and pathogen-infected cells, but are also a major cellular reservoir for HIV-1 infection. HIV-1 is reported to infect macrophages by relatively inefficient processes of macropinocytic and endocytic uptake of cell-free virions. Earlier, we described directed cell-to-cell spread via virological synapses between T cells and from macrophages to T cells, a process of infection more efficient than cell-free uptake. However, the dominant mechanism by which HIV-1 spreads from its principal target, the CD4+T cell, to macrophages is unknown.

## Material and methods

T cell lines and primary CD4+Tcells were infected with various HIV-1 strains, and were subsequently co-cultured with autologous (where applicable) monocyte-derived macrophages. Macrophage infection was qualitatively and quantitatively characterised using conventional and multispectral flow cytometry, confocal and electron microscopy, and detection of viral reverse transcription products by qPCR.

## Results

Co-culture of HIV-1-infected T cells and macrophages rapidly led to detection of Gag and viral (v)DNA in macrophages, which peaked after 3 hours. Separation of macrophages and HIV-1-infected T cells by a virus-permeable membrane significantly decreased macrophage infection, demonstrating that cell-cell contact is essential for T cell-to-macrophage spread of HIV-1. Macrophage uptake of T cell-associated vDNA was not significantly reduced by blockers of viral Env-receptor interactions, viral fusion, or macropinocytosis. However, cytoskeletal paralysis and inhibition of dynaminactivity did significantly decrease HIV-1 +T cell uptake. HIV-1 -infected CD4+ T cells with morphological and phenotypic features of apoptosis and necrosis were selectively phagocytosed by macrophages, resulting in gradual degradation of T cell-associated vDNA. However, productive HIV-1 infection of macrophages took place despite this, suggesting viral escape from degradation. Infectious molecular clones representing transmitted/ founder (T/F) HIV-1 have recently been derived, and were suggested to be non macrophage-tropic. However, although T/F viruses demonstrated low macrophage infectivity in a cell-free form, phagocytosis of T cells infected with these viruses led to efficient macrophage infection.

## Conclusion

We describe a novel mechanism of macrophage infection by macrophage recognition and clearance of dying HIV-1-infected CD4+T cells. Macrophage uptake of dying HIV-1-infected T cells is likely to take place at all times during the natural course of infection. However the highest impact will probably be during acute infection when transmitted virus infects a single, or a small number of permissive cells, forming an initial focus of infection. We predict that the massive apoptosis observed during the first weeks of acute HIV-1 infection in mucosal lymphoid tissue will lead to rapid recruitment of macrophages to engulf the dying cells, which thereby become infected forming a stable local virus reservoir.

